# Hydrocarbons in the Meniscus: Effects on Conductive Atomic Force Microscopy

**DOI:** 10.1021/acs.langmuir.2c03222

**Published:** 2023-03-20

**Authors:** Nathan L. Tolman, Ruobing Bai, Haitao Liu

**Affiliations:** Department of Chemistry, University of Pittsburgh, Pittsburgh, Pennsylvania 15260, United States

## Abstract

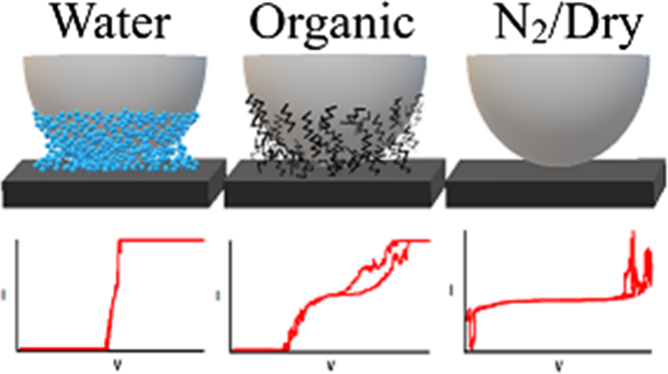

It is commonly accepted that during conductive atomic force microscopy (CAFM) measurement in ambient, a liquid meniscus can form between the tip and the sample. Such a liquid bridge, normally assumed to be composed of water, is a major factor in analyzing and understanding CAFM results. Here, we show that the adsorption of adventitious hydrocarbons from the air to a surface can greatly affect CAFM data both in imaging mode and in local spectroscopy (current–voltage or *I*–*V* curves). We propose a model to explain the phenomena whereby hydrocarbon contaminates contribute to the composition of the liquid bridge between the tip and the sample.

## Introduction

At the time of writing this paper, a quick Google image search of either “atomic force microscopy (AFM) schematic” or “conductive atomic force microscopy (CAFM) schematic” will turn up thousands of pictures of a needle like object resting on a sample connected to a laser and a computer. While this is the true configuration of an atomic microscope in ultrahigh vacuum (UHV), it is not an accurate picture for the most common AFM configuration which is in air. These simple schematics are very misleading to novice AFM users because in air at most humidity levels there is a liquid bridge or meniscus caused by capillary condensation between the probe tip and the sample. This is not such a problem for regular AFM imaging but the importance of this liquid bridge in CAFM cannot be ignored.

First, because the local environment in which AFM experiments are carried out may greatly affect the contact between the probe and sample, the effect of humidity on adhesion and attractive forces will be discussed briefly. It is well known that, above a certain relative humidity (RH) threshold, a capillary water bridge exists surrounding the whole probe tip and extending to the sample surface that is responsible for most of the attractive/adhesion forces between the two. Below the humidity threshold, water is in the vapor phase and any capillary condensation present occurs in the very small asperities (if any) of the probe extending toward the sample.^1^ Hu,^2^ Sedin,^1^ Thundat,^3^ and Xu^4^ have all studied humidity thresholds for liquid bridge formation and increased adhesion on mica and agree that the threshold for that material is about 20% RH. To the best of our knowledge, the humidity threshold for highly ordered pyrolytic graphite (HOPG) has not been published.

**Figure 1:**
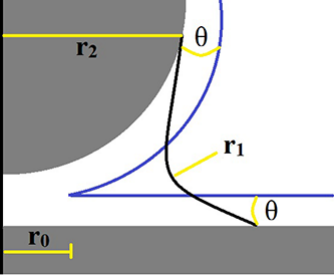
Schematic of the tip-sample interface defining the above variables. Bold horizontal line and the half circle represent the sample and the tip, resepctively. Only the right half of the tip-sample interface is shown.

According to Grobelny and coworkers, the capillary or meniscus force is the dominating adhesion force in nonvacuum environments.^5^ Interestingly and applicable to our work, Sedin studied the effect the water contact angle (WCA) has on adhesion forces. They measured adhesion forces on substrates of varying surface energies and found that adhesion forces decrease with increasing WCA/hydrophobicity.^1^ Grobelny provides the following equations for capillary force (*F*_cap_):
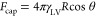
1
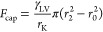
2with Figure 1 defining the variables where *R* is the radius of the probe tip, *r*_0_ is the radius of the meniscus where the adsorption layers overlap, *r*_2_ is the radius at the top of the meniscus, *r*_K_ is the meniscus equilibrium radius obtained from the Kelvin equation,^1,5^ θ is the WCA, γ_LV_ is the liquid–vapor surface tension, and γ_LV_/*r*_K_ is the Laplace pressure within the meniscus that draws the meniscus over r_0_ where the adsorption layers overlap, then π(*r*_2_^2^ – *r*_0_^2^) is the annular projection of the capillary on the surface.^5^Equations 1 and 2 provide a correlation between the WCA and the size of the capillary; specifically, the surface area covered by the meniscus is smaller for surfaces with a high WCA. Sedin tested several surfaces like quartz and mica but for HOPG she used a literature value of 80° which according to Kozbial and coworkers^6^ is an HOPG surface already covered with adventitious hydrocarbons from air. In fact, Sedin attributes her wide range of humidity transition points on contaminations on quartz and mica as well as differences in probes.

Also applicable to our work is Sumaiya and coworkers’ publication on improving the reliability of contact resistance measurements using CAFM.^7^ They found differing conductance as large as an order of magnitude in ambient air on HOPG. The reasons they list include changes in chemistry, surface structure, environmental conditions, contamination, and material characteristics. To counter these issues, they used diamond tipped probes, an N_2_ environment, and heating of the HOPG. They found that the most significant factor in reducing the standard deviations of their measurements was the switch from ambient air to N_2_ suggesting that humidity is a major factor in CAFM data quality.

Lanza and coworkers in two papers^8,9^ studied the conductivity images of a high-*k* dielectric thin film. They showed in their conductivity images that the resolution improves in an argon environment with the absence of water. They observed increased currents with 44% RH versus 0.5% RH. Lanza et al. also observed conductivity changes by looking at the slopes of *I*–*V* curves taken at points on the sample in each of three environments: air (45% RH), N_2_, and UHV. He again found that the presence of water greatly increased conductivity.^8,9^

Patel and coworkers showed that airborne hydrocarbon contamination exposure for two and 24 h reduces the conductivity of basal plane HOPG when observed through CAFM.^10^ This work has confirmed Patel’s work except the amount of time required for the change of conductivity to occur in our local environment is 5 to 7 days versus Patel’s 2 or 24 h. Local environments have different humidity levels and different types of hydrocarbon contamination present. Work from our group using ultraviolet photoelectron spectroscopy has shown that not only is hydrocarbon contamination on graphite immediate but it is also constantly changing in nature over at least 20 h of air exposure.^11^

In a CAFM imaging study on the conductivity of HOPG, Banerjee and coworkers showed that the conductivity of the basal plane depends on the height of the HOPG ribbon.^12^ Banerjee also sometimes observed current dipping at the bottoms of step edges and current spiking at the tops of step edges. Note that on the same ribbon edge, the spikes in current were not always observed. Banerjee explained that the increased conductivity of the top sheets was because they are less strongly adhered: during the peeling off process of HOPG exfoliation, the top sheets of HOPG are exposed to more sheering forces and are therefore dislocated more laterally and vertically. This effect lowers adhesion and causes the π-electrons to be less involved in interlayer bonding which allows for better charge-carrier mobility and therefore higher conductivity. In another theory, they also claimed that the top sheets may be crumpled which dopes the zero-gap semimetal with added carriers.^12^ The explanation given for the conductivity dips and spikes at step edges is that for zigzag edges, which Banerjee explains are expected to have more charge carriers present, the conductivity increases, while for armchair edges, the conductivity dips. Banerjee explains that the same ribbon can contain regions of both types of edges due to the mosaicity of HOPG which explains the presence and absence of dips and spikes on the same ribbon.^12^

Shvets and coworkers in 2010 reported similar observations on the conductivity of HOPG as Banerjee; however, the explanations given are vastly different. Shvets argued that the conductivity depends more on the history of the interactions of the probe-sample than on any changes in the surface physical properties. Shvets showed that sharp changes in topography caused sharp changes in conductivity that could last the entire length of a plateau or ribbon and could be seen especially when comparing forward and reverse scans in these areas.^13^ Shvets further explained this connection between topography relief and conductivity jumps. First, he pointed out that in the absence of defects in the scanning field, the conductivity changes were insignificant. Because of this observation, he concluded that some random process occured during the transition of the probe to a higher level in which the probe collected conducting particles of graphene and moved them further along the plateau which increased the electrical conductivity of the probe with the surface. Subsequent changes in relief can also cause the loss of said conducting particles.^13^

While these prior studies offer great insight into the CAFM characterization of HOPG, they all have their limitations. Lanza did not explore the effects hydrocarbons may have had on their samples. In the work of Lanza, the adsorbed hydrocarbons would have to be somewhat polar to adsorb to the oxide. Patel did not go into the effects that water may have had in conjunction with hydrocarbon contamination. Neither Banerjee nor Shvets considered water or hydrocarbons as the source of the conductivity landscape observations across HOPG ribbons. Shvets blamed graphene flakes collected from the sample for the observed change of conductivity; one may conjecture that the role of graphene flakes may very well be played by water or hydrocarbons adsorbed on HOPG.

Both current-map images and local spectroscopy (*I*–*V* curve graphs) were used to electrically characterize a sample having variable regions of resistance. For example, Douhéret and coworkers studied the nanoscale electrical characterization of organic photovoltaic blends by CAFM. They used CAFM imaging to illustrate electrical phase separations as well as total current variation and local spectroscopy (*I*–*V* curves) to determine material resistivities, barrier heights, carrier mobilities, and power efficiencies.^14^ However, they did not mention, like most CAFM studies, the liquid bridge crossing the interface between the probe and the sample and therefore do not consider the effects it could have had on their data, especially on the variation of the current.

**Figure 2:**
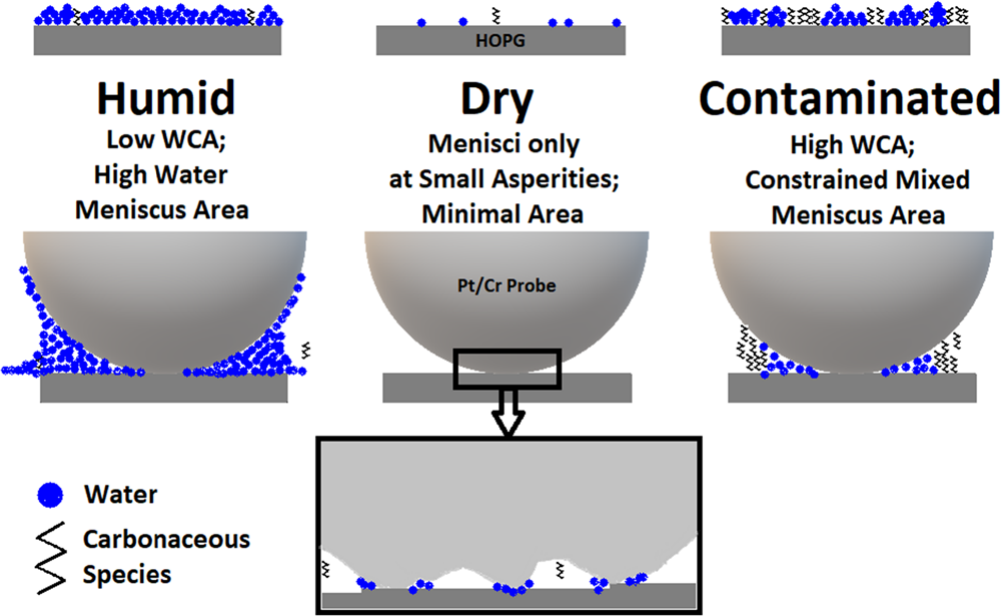
Liquid capillary bridge schematic between the probe tip and sample. Humid and dry conditions imply that the HOPG has been freshly exfoliated and the time required to land the probe on the sample has been minimized as much as possible. Contaminated refers to a sample that was either purposefully contaminated with hydrocarbon after exfoliation or aged in air to collect adventitious hydrocarbons before landing of the probe.

Here, we show that the addition of hydrocarbons to the sample, either through intentional exposure or simple air exposure, affects CAFM imaging and local spectroscopy data. We show that adsorption of adventitious hydrocarbon from air contributes to the liquid bridge between the conductive probe and the sample and significantly impacts the CAFM data in both current imaging and spectroscopy modes (Figure 2). Specifically, in low humidity conditions, airborne hydrocarbons alone can form the liquid bridge and increase the tip-sample conductance; however, under high humidity conditions, airborne hydrocarbons reduce the contact angle and consequently reduce the conductance.

## Experimental Section

### Materials

HOPG (SPI-2 grade, 10 × 10 × 2 mm) was purchased from SPI Supplies Inc. and was used for all experiments. AFM probes (silicon ContE-G with a Cr/Pt conductive coating, 13 kHz resonance, and 0.2 N/m) were purchased from Budget Sensors, Inc. All-platinum AFM probes (12Pt400B, 0.3 ± 40% N/m) were purchased from Rocky Mountain Nanotechnology, Inc. 1-Tetradecene (GC, ≥97%) and 1-octadecene (GC, ≥95%) were purchase from Sigma Aldrich Inc. Naphthalene (Reagent Grade Flakes) was purchased from Fisher Science Education. Scotch tape was purchased from 3M, Inc. Nitrogen (Ultra High Purity, 99.999%) was purchased from Matheson Gas, Inc.

### CAFM Imaging

All AFM experiments were carried out on an Asylum MFP-3D atomic force microscope. For all conductivity images shown below, CAFM contact mode was used with a setpoint applied force of 8.9 nN, scan rate of 0.5 Hz, scan size of 5 μm, an applied voltage of −61 mV (voltage that eliminates the most noise), and a 1 MOhm resistor was manually added in serial with the sample to limit the current. For data processing, the same parameters were used for every conductivity image with bias applied to the tip (Mud color scheme, 4 nA range).

### Local Spectroscopy (*I*–*V* Curves)

No added resistor to the circuit was used for spectroscopy. All local spectroscopy was done with the feedback mechanism turned on to ensure a constant tip-sample interface distance. Setpoint applied force was set to the default for contact mode (18 nN for the Cr/Pt silicon probes and 27 nN for the all Pt probes). Current sense was also left at the default of 200 pA/V. Local *I*–*V* curves were then taken at various times from exfoliation. Local *I*–*V* curves were from 2 to −2 V at 8 V/s (1 s total collection time).

### HOPG Exfoliation, Aging, and Positive Controls

The Scotch tape method was used for all exfoliations. The HOPG was only handled with stainless steel tweezers. Unless stated otherwise, air aging was carried out with HOPG in a glass vial sitting on its side to minimize the deposition of particulate matter and aged in a chemistry laboratory. For positive controls, HOPG was first exfoliated and then placed in a closed petri dish with four partitions in which one partition contained the contaminant reservoir while the freshly exfoliated HOPG was stored in the opposite partition for 10 min.

## Results and Discussion

### Force–Distance–Current Plots To Establish the Presence of an Organic Meniscus

**Figure 3:**
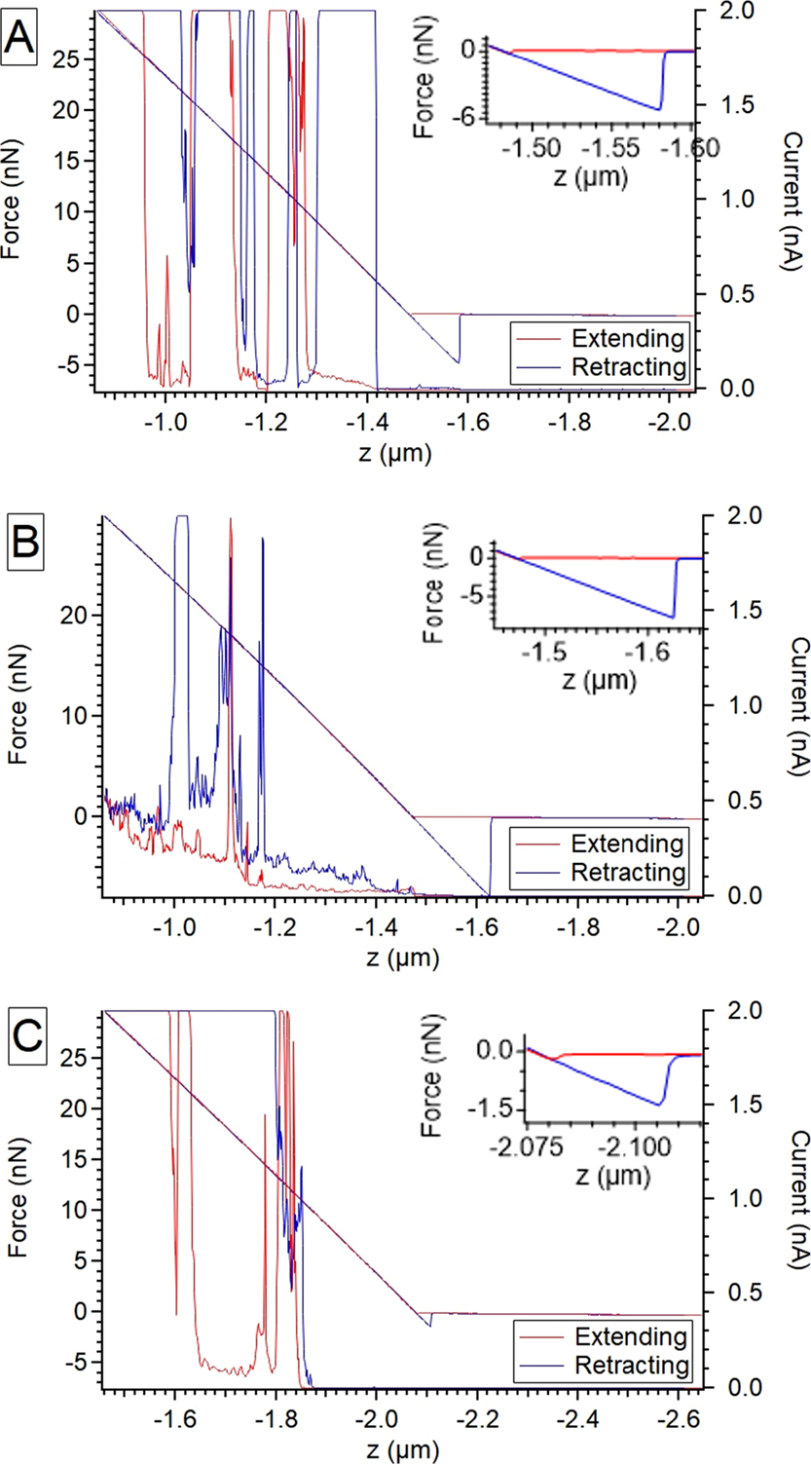
FDC plots support the presence of a meniscus formed by adsorbed airborne-hydrocarbons on HOPG. All data were collected with a 50 mV bias applied to the sample. All-metal (Pt) probes were used in all cases. (A) Air aged HOPG measured in air, (B) sample (A) at 5% RH purged with N_2_, (C) sample (B) after exposing to 1-tetradecene vapor, under 5% RH in N_2_.

In order to show that a probe-sample interface meniscus can exist in an organic phase, force–distance–current (FDC) plots were collected using an all-platinum AFM probe. The results are shown in Figure 3. A sample of HOPG was allowed to air-age for more than a month ensuring that the sample surface was covered with both airborne hydrocarbon contaminants and water from the environment. Figure 3A shows the FDC plot of this air-aged contaminated sample. As can be seen, the current fluctuates between zero and saturation several times during the collection of the FDC data, which takes about 2 s. The sample and the AFM box were purged with N_2_ until 5% RH was reached (about 10 min). The FDC plot was acquired again (Figure 3B), which shows large decreases in the current, presumably due to the removal of adsorbed water due to purging by dry N_2_. The sample was then subjected to 1-tetradecene vapor. It can be seen in Figure 3C that the current again reached saturation at high force values but blocking behavior was also observed during tip retraction. Earlier work by O’shea et al. suggested that such blocking behavior may be attributed to the presence of tip coating damage or even removal.^15^ In our experiment, this scenario is ruled out since the probe is entirely platinum. The other cause of blocking behavior is liquid contamination in the probe-sample interface. In this case, the only liquids present were presumably organic contaminants remaining on the sample and condensed 1-tetradecene vapor. Organic liquids are likely less conductive than water resulting in a large increase in resistance at the tip-sample interface from the presence of organics alone. Also, the presence of organics would affect the geometry of the meniscus as shown in eqs 1 and 2 above. An increase in organics would result in a smaller meniscus footprint and therefore less current as well.

Close-ups of the point of contact in the force–distance curves can be found in Figures S1–S3. The change in the adhesion force between the tip and sample, or the force required to pull the probe tip free of the substrate, suggests a significant change of the meniscus in response to the changes of the environment. Calculated from the force–distance curves at the point of contact (insets in Figure 3), the three values for (A), (B), and (C) were 7.8, 4.4, and 1.4 nN, respectively. We suggest that the significant decrease is due to changes in the meniscus composition and/or geometry. The meniscus composition change can be tracked through the capillary forces listed above. Under 5% RH, after the addition of 1-tetradecene vapor, the capillary force is greatly reduced but still adhesion remains suggesting that a meniscus is still present. The significant drop in the magnitude of the force suggests the remaining meniscus is likely organic and/or mostly 1-tetradecene.

Overall, the data suggest the presence of a liquid meniscus between the AFM tip and a HOPG surface contaminated by airborne hydrocarbons, even under 5% RH in N_2_. The changes in the current and adhesion force after introducing 1-tetradecene seem to suggest an equilibrium in the meniscus composition, i.e., airborne contaminants on the surface seem to have been, at least partially, replaced by 1-tetradecene. Additional work is needed to fully understand the characteristics of the FDC plots.

### Local Spectroscopy (*I*–*V* Curves)—Positive Controls (Organic Contamination of HOPG)

**Figure 4:**
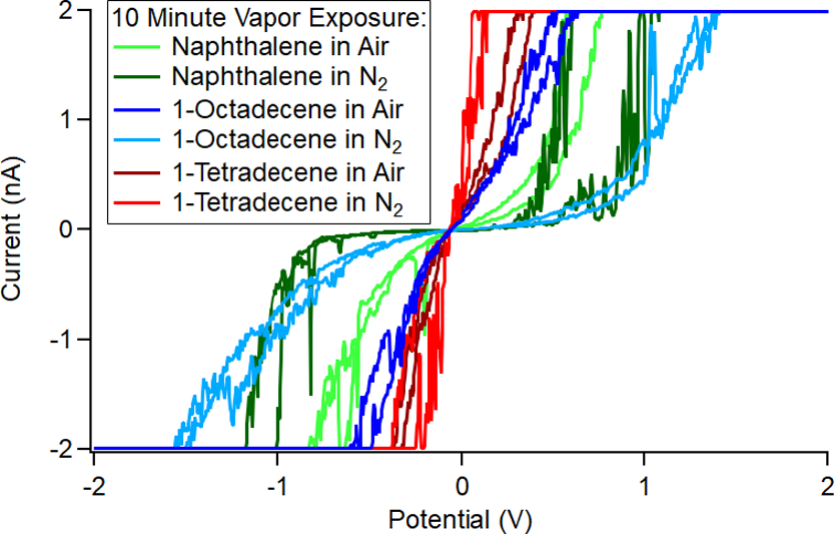
*I*–*V* curves using Cr/Pt coated silicon probes on the HOPG basal plane exposed to (greens) naphthalene, (blues) 1-octadecene, and (reds) 1-tetradecene vapors in both 43% RH air and 1.5% RH N_2_.

**Figure 5:**
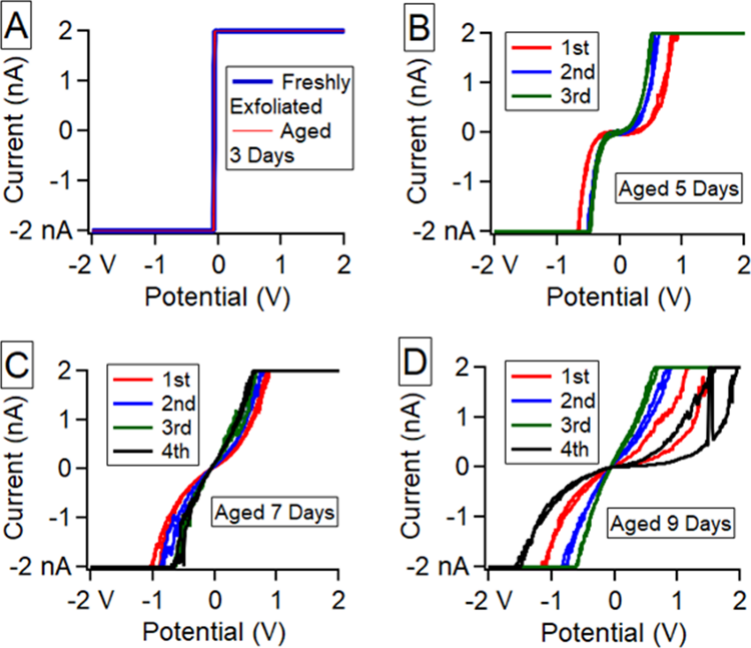
*I*–*V* curves using Cr/Pt coated silicon probes (legends show sequence of repetitions) on HOPG in air immediately after HOPG exfoliation (A), 3 days aged (A), 5 days aged (B), 7 days aged (C), and 9 days aged (D). Consecutive curves were taken in (B–D).

Positive control experiments were carried out where freshly exfoliated HOPG was exposed to hydrocarbon vapors known to adsorb well to HOPG for 10 min. As can be seen in Figure 4 below, for the three hydrocarbons used, (green) naphthalene, (blues) 1-octadecene, and (reds) 1-tetradecene, in all cases, the conductivity shown in the *I*–*V* curve was reduced compared to the freshly exfoliated HOPG in air (Figure 5A below). Since the effect is different for each hydrocarbon, this shows that the composition of the liquid bridge can greatly affect the results of the CAFM local spectroscopy experiment. For example, the sample exposed to 1-octadecene in N_2_ is poorly conducting but the conductivity greatly improves in air, presumably due to the presence of water vapor. The main point to be taken away from this experiment is that the addition of hydrocarbons to the liquid bridge reduces conductivity of local spectroscopy in CAFM.

### Local Spectroscopy (*I*–*V* Curves)—Clean versus Aged HOPG and Dry versus Humid Environment

We studied the evolution of the HOPG basal plane *I*–*V* curve shape with air aging time which results in hydrocarbon adsorption and increased WCA.^6^ Similar work has been reported by Patel and coworkers,^10^ but unlike Patel, we observed the highest possible conductivity (saturation of the current amplifier) in Figure 5A for 4 days; starting on the 5th day (Figure 5B,) the conductivity began to show a decline and not until the 9th day (Figure 5D) in this case was the decline significant. This experiment was carried out with the same result 3 times each (6 total) for two different researchers. The conductivity of these six HOPG samples all showed an initial highly conductive state and then a decline was observed in 5–7 days. In Patel’s work, they observed a change in conductivity both at 2 and 24 h.^10^ This difference in the kinetics is not surprising considering hydrocarbon concentration and type can vary from place to place. RH may play a role in protecting the graphite from contamination as well.^16^

It should be noted that the amount of time to collect an *I*–*V* curve in all of the above cases is 1 s. When the voltage was applied for a much longer period of time, such as during a chronoamperometry experiment, the current exhibits quite different behavior which tends toward a maximization of current over time in most cases. Details of chronoamperometry experiments are shown in the supplementary information (Figures S4–S7). Each chronoamperometry experiment except the freshly exfoliate HOPG case, within the 1 s time frame, starts at zero current and time is required to change the current significantly. Only freshly exfoliated HOPG in humid air shows an immediate jump to maximized conductivity; this phenomenon can be seen in *I*–*V* curves shown in Figure 5A as well. In fact, the chronoamperometry experiments may even show the effect of changes in the liquid bridge over time due to such factors as diffusion of molecules across the HOPG surface, electrostatic attraction/repulsion, or local heating.

By linking Sedin’s^1^ work with Lanza’s,^8^ we can conceive of a model for the behavior observed shown here for CAFM local spectroscopy. Upon landing of the probe, a liquid bridge is immediately formed from local species on the sample in an area roughly several hundreds of nm^2^, in the air through capillary condensation, and already on the probe. The nature of this mixture affects the liquid bridge geometry because the increased WCA from hydrocarbons^6^ results in decreased radius of the liquid bridge footprint on the sample, (eqs 1 and 2), resulting in decreased conduction area and lower conductivity and vice versa.^1,8^

However, these experiments cannot exclude the possibility of tip wear (these experiments used Cr/Pt coated Si tips)^15^ or the accumulation of an organic contamination layer (but not a meniscus) on the surface, both of which would result in increased resistance across the interface regardless of the presence of a liquid bridge connecting probe to surface. This is because tunneling across sections of the probe missing the conductive coating or tunneling through a layer of organics would innately have increased resistance to current flow.

**Figure 6:**
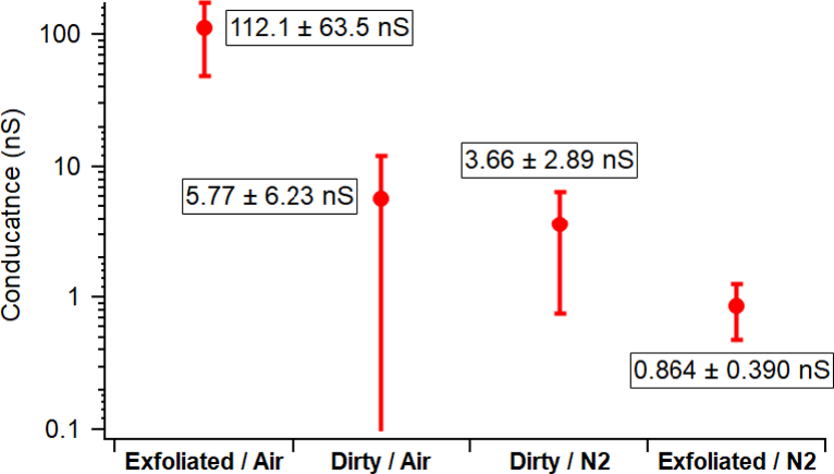
Plot of conductance from *I*–*V* curves using Cr/Pt coated silicon probes on HOPG in four different conditions.

**Figure 7:**
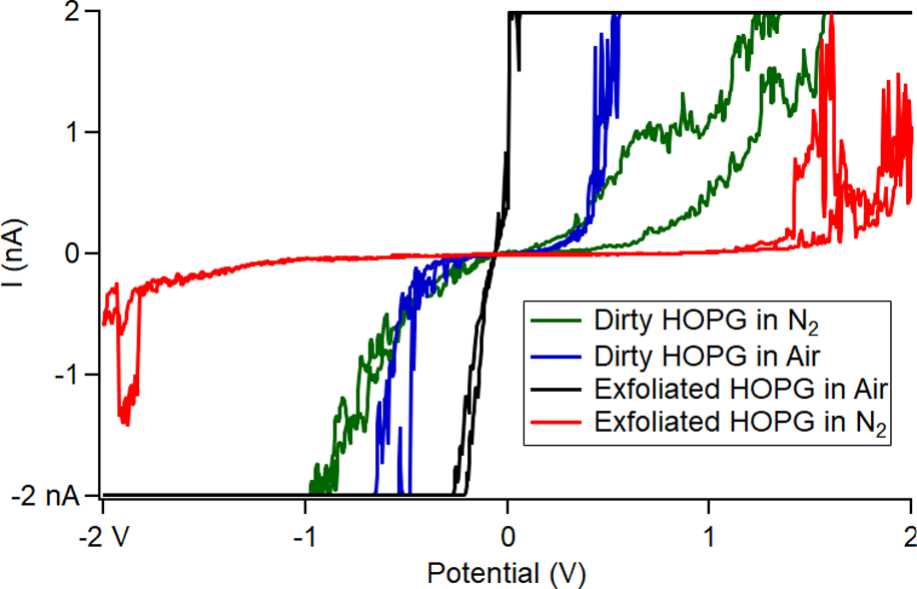
*I*–*V* curves using Cr/Pt coated silicon probes on exfoliated and five-week aged HOPG in air (22% RH) and N_2_ (0.5% RH).

Next, we did a similar experiment to study the effect of humidity on the conductivity. Using an HOPG sample that was extensively contaminated by airborne hydrocarbon (5-weeks aged), we collected *I*–*V* curves in 28% RH air versus 0.5% RH N_2_ and compared them. The conductivity (shown in Figure 6) of this air-contaminated HOPG sample is similar, because of the magnitude of the error, with and without water, 5.77 ± 6.23 nS, at 28% RH versus to 3.66 ± 2.89 nS at 0.5% RH. For clean, exfoliated HOPG, the conductivity in 0.5% RH N_2_ was 0.864 ± 0.390 nS while the conductivity in 28% RH air is 112.1 ± 63.5 nS which is almost at the detector maximum. This experiment was repeated three times to confirm its reproducibility with approximately 15 measurements in N_2_ taken for dirty and exfoliated HOPG. Sample *I*–*V* curves are shown below (Figure 7) while the data used for the calculation of the conductance are provided in the supplementary information (Figures S8–S11). This experiment illustrates the importance of the liquid bridge and its composition for CAFM and fits the model described above. The addition of water or increase in RH with or without hydrocarbons present seems to increase conductivity overall. Our data also show that in dry N_2_, an airborne-contaminated HOPG may show higher conductance than a clean HOPG (Figure 6 and green vs red in Figure 7), although the statistical difference between the two data sets is not completely outside their error bars. These data, combined with those from force–distance curve measurements, suggest that airborne hydrocarbon alone can form a liquid bridge in the absence of water and impact the tip-sample conductivity.

The experimental results in Figures 6 and 7 and the CAFM landscape images were obtained with silicon probes coated with conductive Cr/Pt. In these cases, while Patel’s^10^ method was used to check for maximum conductivity of the probe on exfoliated HOPG, it is possible that tip wear or damage to the conductive coating could have caused the same loss of conductance as mentioned by O’shea.^15^

Note that previous studies reported a much higher conductivity (lower resistance) for freshly exfoliated HOPG using CAFM local spectroscopy.^7,15,17^ Zade and coworkers in particular reported conductivity orders of magnitude larger than we do, however, they first showed that this highly conductive state does not occur for several seconds to a few minutes. Upon landing the probe, they reported very low conductivity with current in the noise level, <100 pA and >50 MΩ, until a jump to high conductivity occurs after a variable time period. They plotted resistance versus time illustrating these current jumps and proposed the fluctuations in current arise from the competition between the formation and removal of oxide on the gold AFM tip. We propose an alternative mechanism that fits with our model where under dry nitrogen we find very low conductivity. After several seconds to minutes, with the nitrogen closed off and the RH gradually rising, we also observe a sudden current jump to high conductivity which is shown in Figures S12 and S13 in the supplementary information. The data for these figures were also collected with a probe that was made entirely of platinum to ensure that the effects we observed were not due to defective CAFM probe platinum coatings.

### CAFM Imaging—Clean versus Dirty HOPG and Dry versus Humid Environment

**Figure 8:**
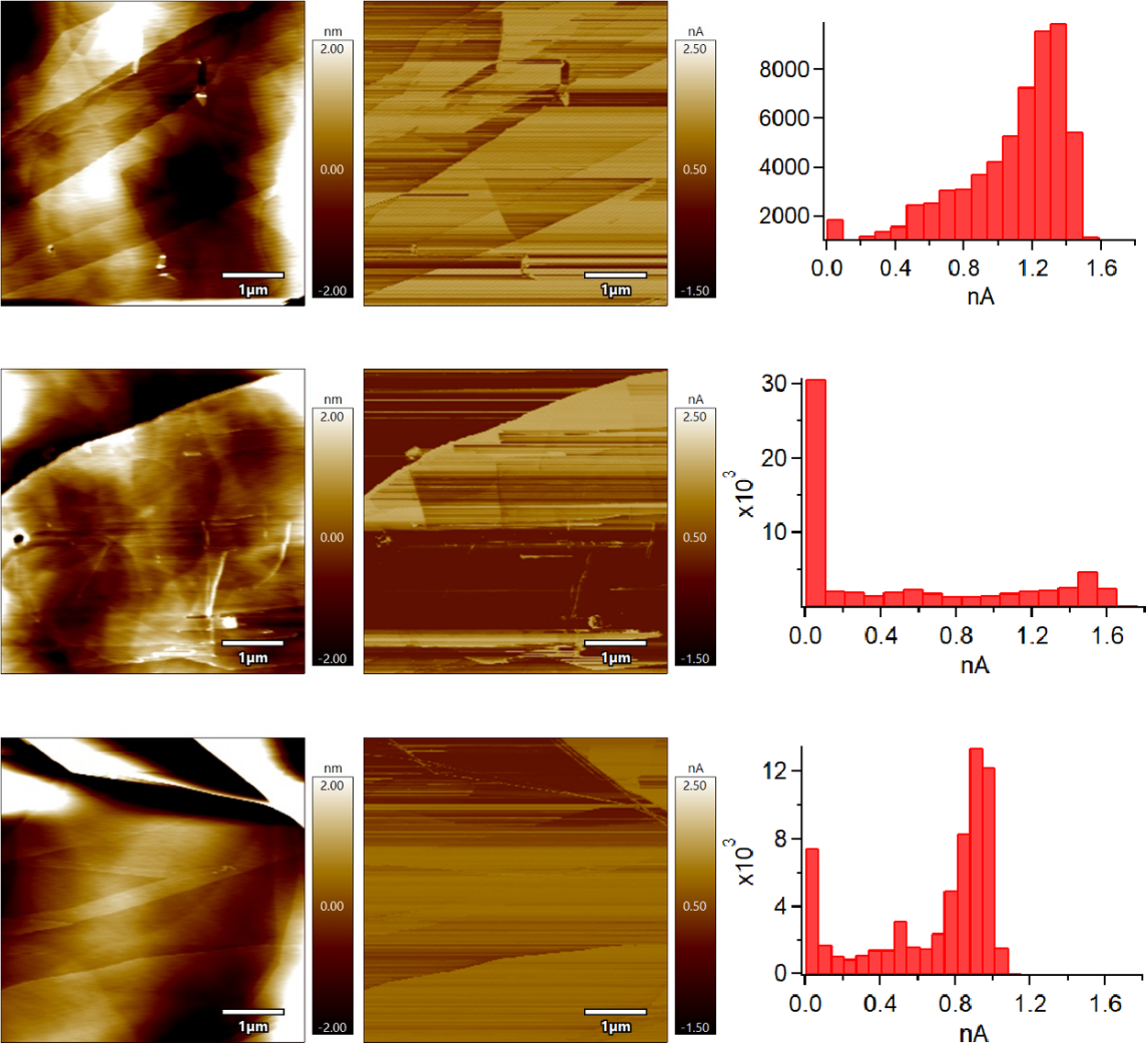
Topography image (left), CAFM image (center), and CAFM image histogram (left) of exfoliated HOPG in air at RH 23% (top), in air 43% RH (middle), and N_2_ at 1.5% RH (bottom). All data were collected using Cr/Pt coated silicon probes.

For imaging in CAFM, our model needs to be slightly altered from that of local spectroscopy because the probe is rastering the surface and is therefore subjected to a constantly changing local environment. As a result, the changes in the data are less noticeable in some cases and require a histogram to be seen. Banerjee^12^ and Shvets,^13^ as mentioned in the introduction, reported CAFM imaging behavior on HOPG which include differences in conductivity between HOPG ribbons, streaking, spiking, and changes in conductivity with drastic changes in topographical relief. We were able to reproduce their observations as can be seen in some of the images below (Figure 8 through Figure 10). The two authors, however, have different explanations for the imaging behavior. Shvets believes that graphene particles are picked up with increasing conductivity and then scraped off on topographical changes or deposited on the basal plane decreasing conductivity. Banerjee’s explanation of the graphite step edge type changes and looseness of ribbons causing drastic changes in conductivity during imaging is sample specific and does not focus on the tip-sample interface as ours or Shvets’ model does. Neither author addresses the liquid bridge but our model does and is more similar to Shvets. Since the local environment is constantly changing during rastering, the meniscus would be constantly encountering new and different species adsorbed to the HOPG surface and therefore would become a constantly changing resistor.

We conducted CAFM imaging experiments of both clean and air-contaminated HOPG in differing RH conditions. Here, 23 and 43% RH samples were freshly exfoliated in air with imaging beginning within 1 min or less after exfoliation so the most highly concentrated adsorbate on the HOPG should be water. In the N_2_ case, air-exfoliated HOPG was placed in the AFM box and filled slowly with dry nitrogen for 20 min to 5% RH or lower with the probe not resting on the sample.

In the case of conductivity imaging, which was studied by Lanza^8,9^ and briefly by Patel,^10^ it can be seen from Figure 8 that current distributions seen in the CAFM images and histograms vary greatly depending on where the image was taken. This agrees with the model we are promoting where the liquid meniscus is a variable resistor changing in composition, drastically in some cases, as the probe rasters the surface. Observations of streaking and spiking made by Banerjee and Shvets can be seen in all of the figures. The histograms show a significant difference in maximum current achieved depending on the RH. While it is not noticeable in the CAFM images but appears in the histograms, Figure 8 top and middle rows have obviously higher maximum current than the dry environment of Figure 8 bottom. Note that the maximum current achieved is reduced by 1/3 or more in the histograms when water is removed from the environment (Figure 8 top and middle rows versus bottom row).

**Figure 9:**
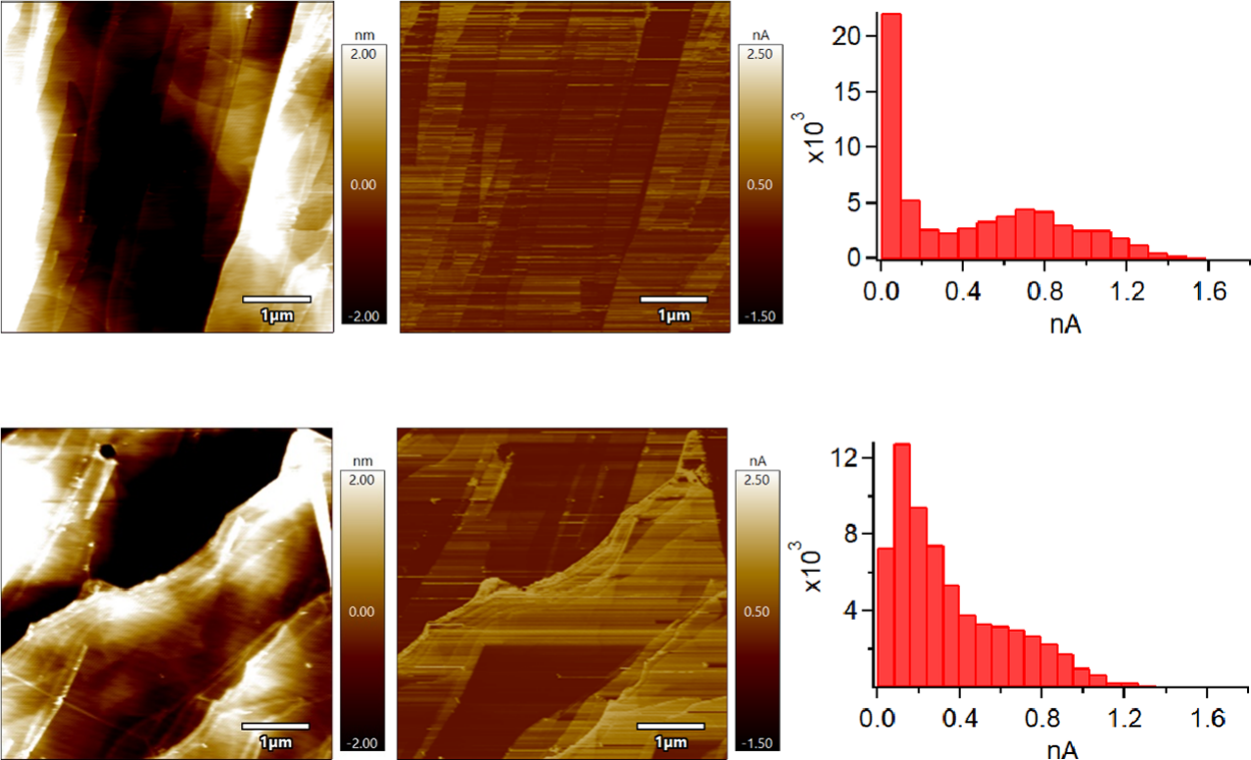
Topography images (left column), CAFM images (middle column), and CAFM histograms (right column) of HOPG aged in air for 24 h (top row) in N_2_ at 1.5% RH and (bottom row) in air at 43% RH. All data were collected using Cr/Pt coated silicon probes.

Figure 9 shows that 24 h of aging in air (random hydrocarbon adsorption) results in a decline in current especially in the absence of water (Figure 9 top row, notice the absence of a peak in the high current regime).^10^ This result confirms Patel’s CAFM imaging study in which 24 h of air aging or exposure to adventitious hydrocarbon contamination causes a decline in conductivity of the HOPG basal plane. With Sedin’s work^1^ in mind, the higher ratio of organics should increase the WCA of the HOPG according to Kozbial et al.^6^ which would decrease the radius of the liquid meniscus, according to Sedin^1^ and Grobelny^5^ and result in a smaller conduction area according to Lanza^8^ and therefore lower current overall. In Figure 9, the presence of hydrocarbon has an equalizing effect with and without water. While not as obvious in the imaging mode (exfoliated HOPG in Figure 8 vs dirty HOPG in Figure 9), as mentioned above in the local spectroscopy section, the presence of hydrocarbon increases conductivity compared to little to no liquid bridge presence at all (green scan vs red scan in Figure 7).

**Figure 10:**
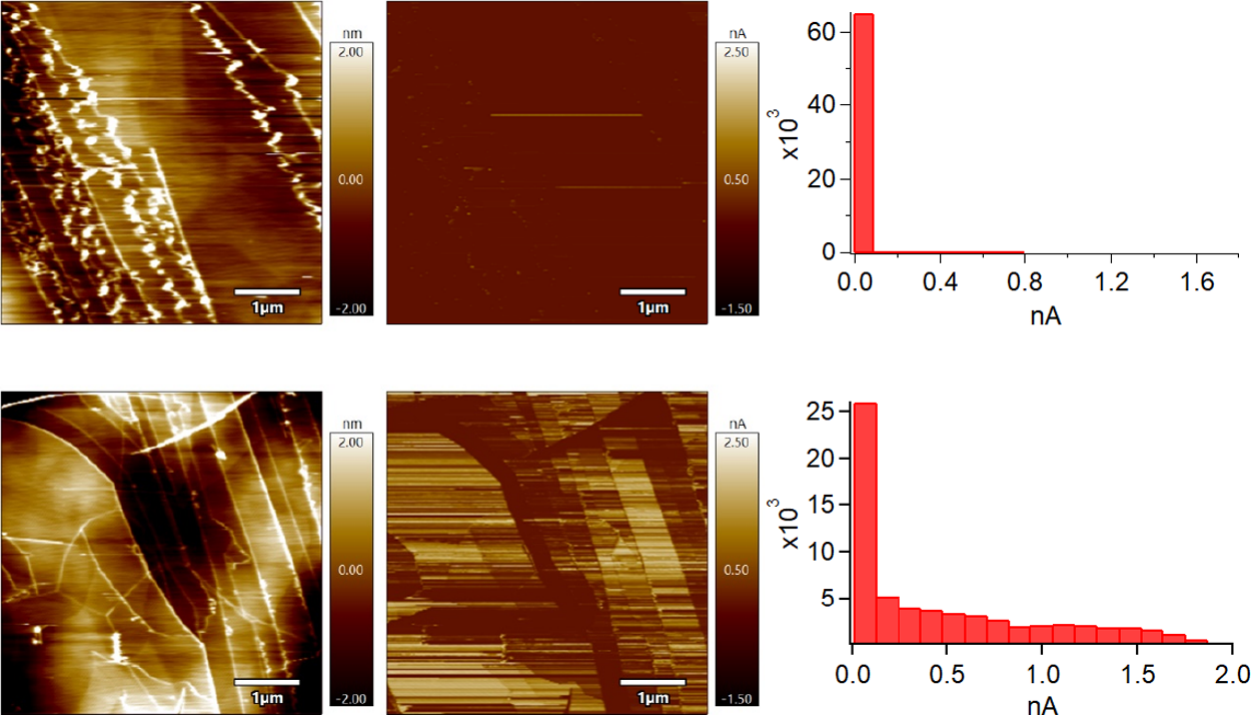
Topography images (left column), CAFM images (middle column), CAFM image histograms (right columns) of HOPG aged in air for 3 weeks and characterized in N_2_ at 1.5% RH (top row) and in air at 33% RH (bottom row). All data were collected using Cr/Pt-coated silicon probes.

According to Salim and coworkers, the nature of the hydrocarbons that adsorb to the surface over time evolves.^11^ The same CAFM imaging trend can be seen with highly contaminated HOPG (aged 3 weeks in air) as shown in Figure 10 but the effect is much more noticeable (Figure 10 top row versus Figure 10 bottom row). The larger number and different nature of hydrocarbons due to long term exposure to adventitious hydrocarbons in air causes a significant dampening of the conductivity landscape of HOPG, noticeable even in the CAFM images, especially in a very low RH environment (Figure 10 top). At high RH%, the contrast is greater too when comparing the change in Figure 9 top row versus Figure 9 bottom row with Figure 10 top row versus Figure 10 bottom row. In Figure 10, the maximum current achieved is most noticeable than all other cases with and without water in the histograms.

It should be noted that the top CAFM image in Figure 10 shows very little detail because all parameters (data collection and imaging processing) for every image were intentionally kept the same. Applying additional imaging processing could bring out more detail in the blander image but processing every image with the same parameters was of greater importance in this experiment.

We also noticed that the reproducibility of the imaging experiments is not as good as the local spectroscopy experiments. We believe this is due to the fact that the changing meniscus is completely unpredictable from sample to sample and from scan to scan, depending on what is found on the sample surface.

## Conclusions

The work discussed above supports a model whereby the liquid bridge between the CAFM probe and the sample is impacted by not only RH but also adventitious airborne contamination. In this model, airborne hydrocarbons play two roles. First, in high RH environments, they affect the WCA of the sample, reduce the radius of the aqueous meniscus, reduce the area of conduction between the sample and the probe, and ultimately, reduce current flowing through the CAFM circuit. In addition, the hydrocarbons are also possible components of the liquid meniscus and impact the conductivity directly. This can be seen in the local spectroscopy experiments, where we found that the presence of adsorbed airborne hydrocarbons increased the conductivity at low RH% but decreased the conductivity at high RH%. Conductive imaging experiments in all cases showed increased current in the presence of water, while the addition of hydrocarbons had an equalizing effect on the current with or without water except for an HOPG surface heavily contaminated from 3 weeks of aging in air.

## Supporting Information Available

Chronoamperometry experiments and figure data from which conductivities were calculated (PDF)
