# Supplementary material for: Hydrocarbons in the Meniscus: Effects on Conductive Atomic Force Microscopy
Source: Langmuir. 2023 Mar 20;39(12):4274–81. doi: 10.1021/acs.langmuir.2c03222 (PMC10061924; doi:10.1021/acs.langmuir.2c03222)
Supplement: Supplementary file 1 — la2c03222_si_001.pdf [file la2c03222_si_001.pdf]

Hydrocarbons in the Meniscus: Effects on Conductive Atomic Force Microscopy

Nathan L. Tolman[a], Ruobing Bai[a], and Haitao Liu[a]*

[a] University of Pittsburgh, Department of Chemistry, Pittsburgh, Pennsylvania, 15260, USA.
*Corresponding author. Tel: +1-412-624-2062. E-mail: hliu@pitt.edu (Haitao Liu)

Supplementary Information

Force-Distance Curves

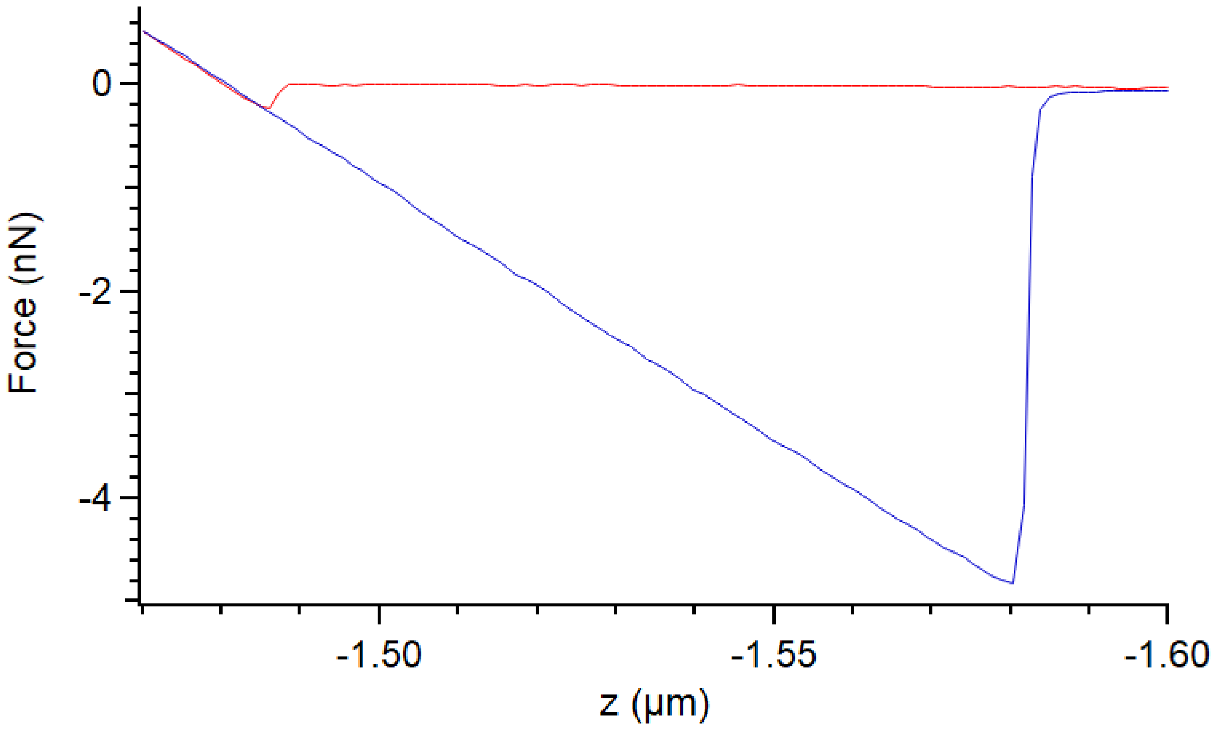

Figure S1. Force-Distance curve enlarged at probe-sample contact for Pt probe on contaminated HOPG in ambient environment.

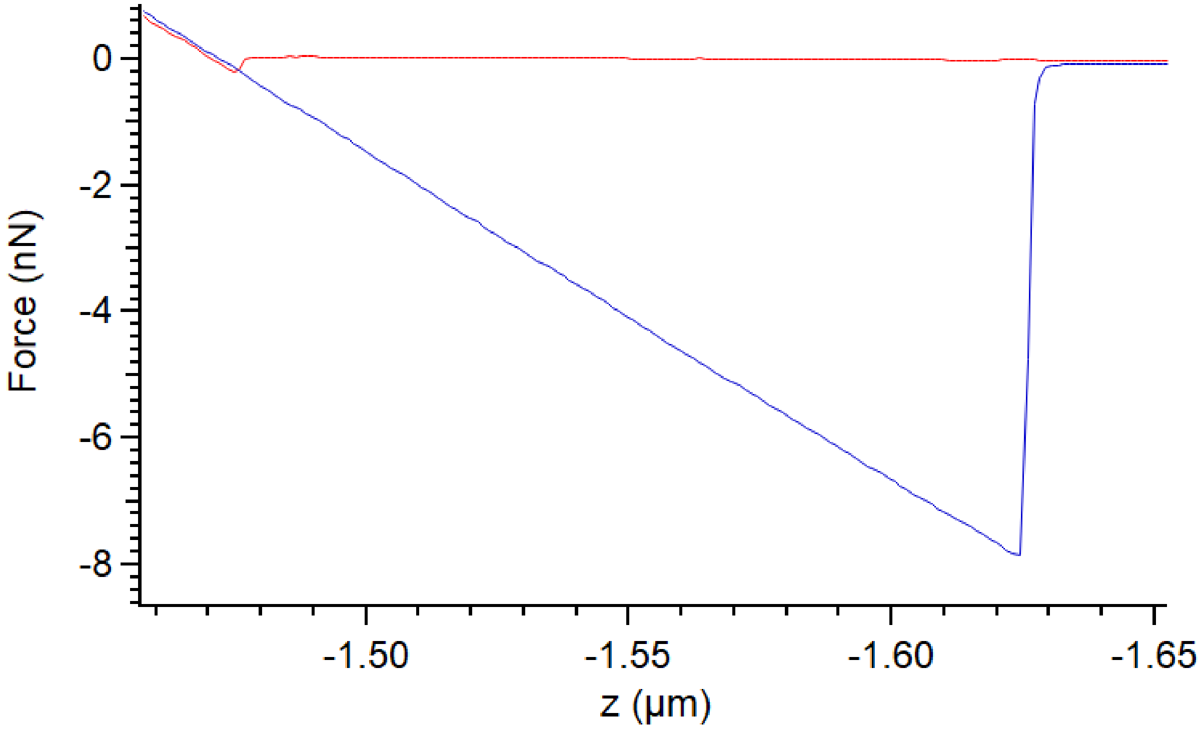

Figure S2. Force-Distance curve enlarged at probe-sample contact for Pt probe on contaminated HOPG in N_2 at 5% RH.

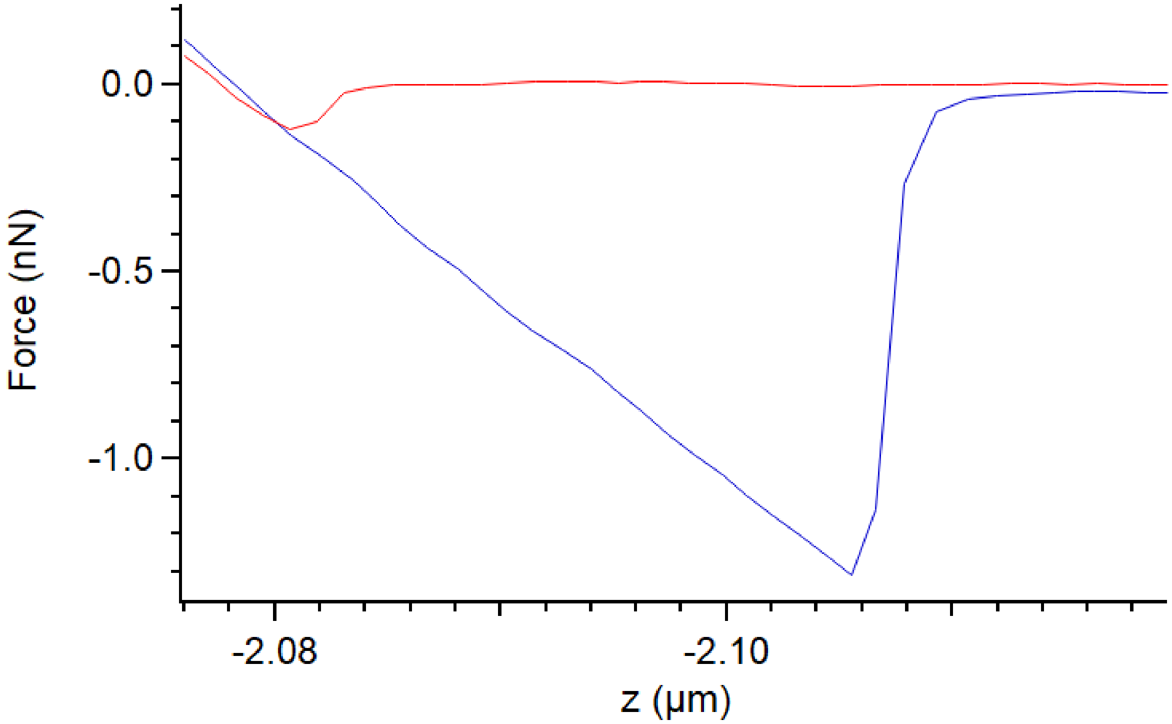

Figure S3. Force-Distance curve enlarged at probe-sample contact for Pt probe on contaminated HOPG in N_2 at 5% RH after the addition of 1-tetradecene vapor to the substrate.

Chronoamperometry Experiments

Chronoamperometry experiments were performed with the conductive platinum probe not in contact with the HOPG surface but a potential of 400 mV already applied to the open circuit. Landing of the probe closes the circuit and the potential is instantaneously applied.

Current is then recorded for one hour in the various environments described below.

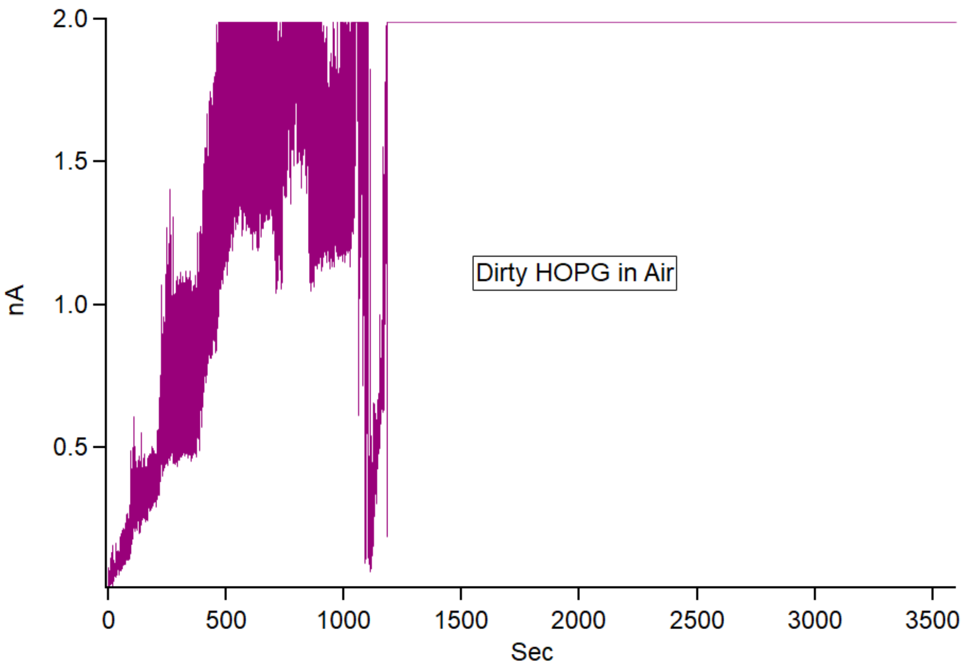

Figure S4. HOPG exfoliated and aged in air for 24 hours. Experiment carried out in ~35% RH air. All data was collected using Cr/Pt coated silicon probes.

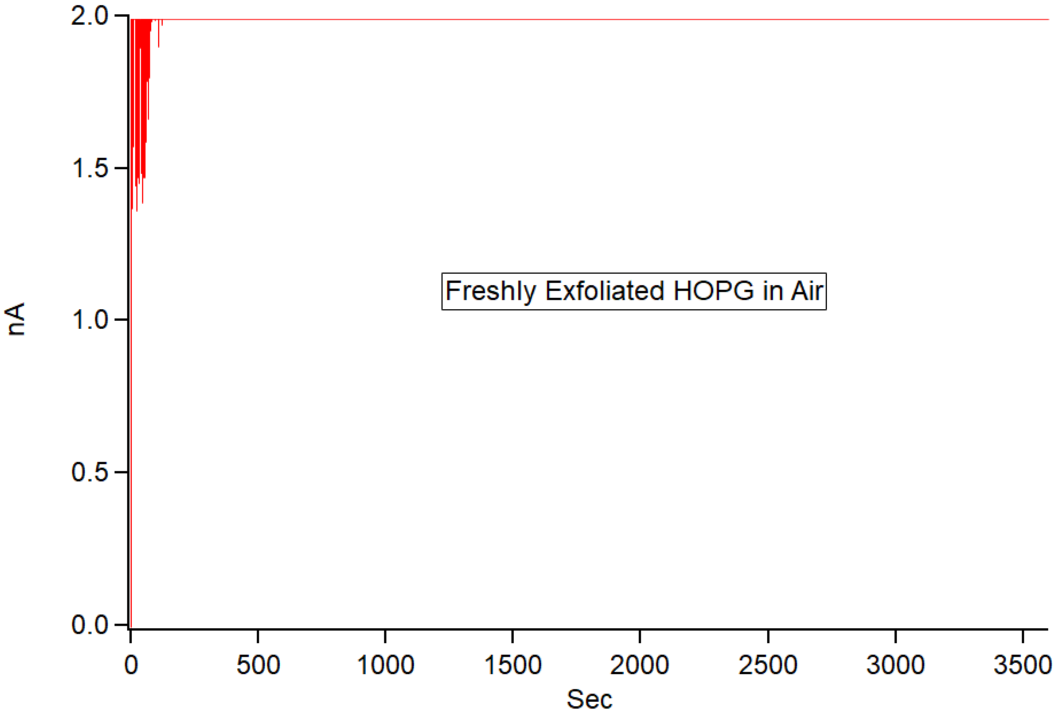

Figure S5. Freshly exfoliated (<1 minute to landing) HOPG in ~35% RH air. All data was collected using Cr/Pt coated silicon probes.

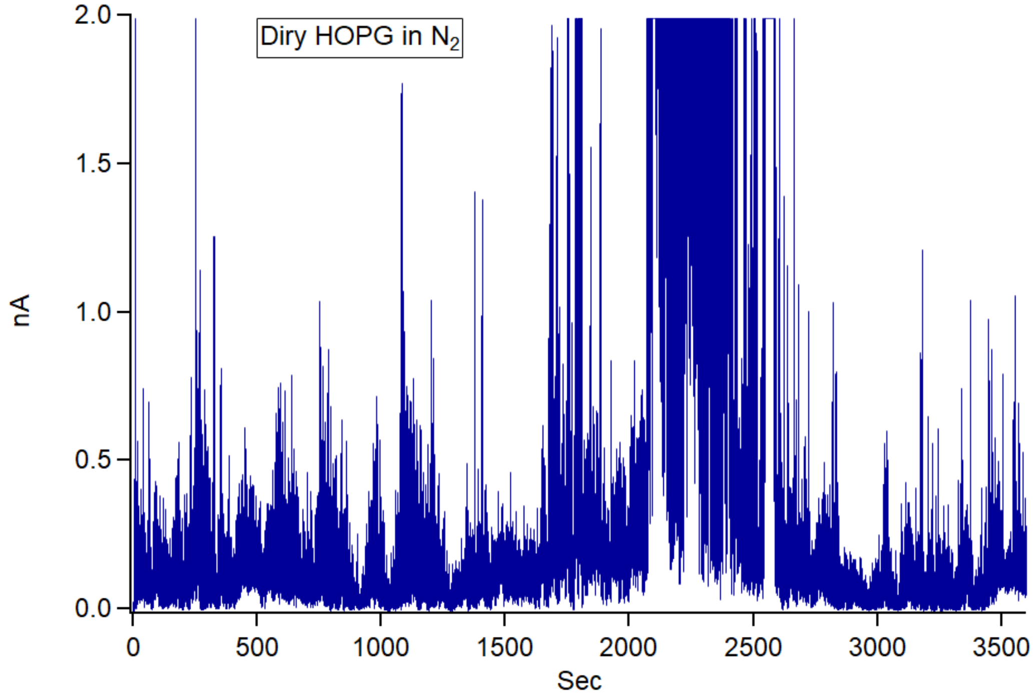

Figure S6. Exfoliated HOPG aged in air for 24 hours then placed in the AFM box which was pumped full of dry nitrogen for about 20 minutes to RH <5% with the probe off of the sample. All data was collected using Cr/Pt coated silicon probes.

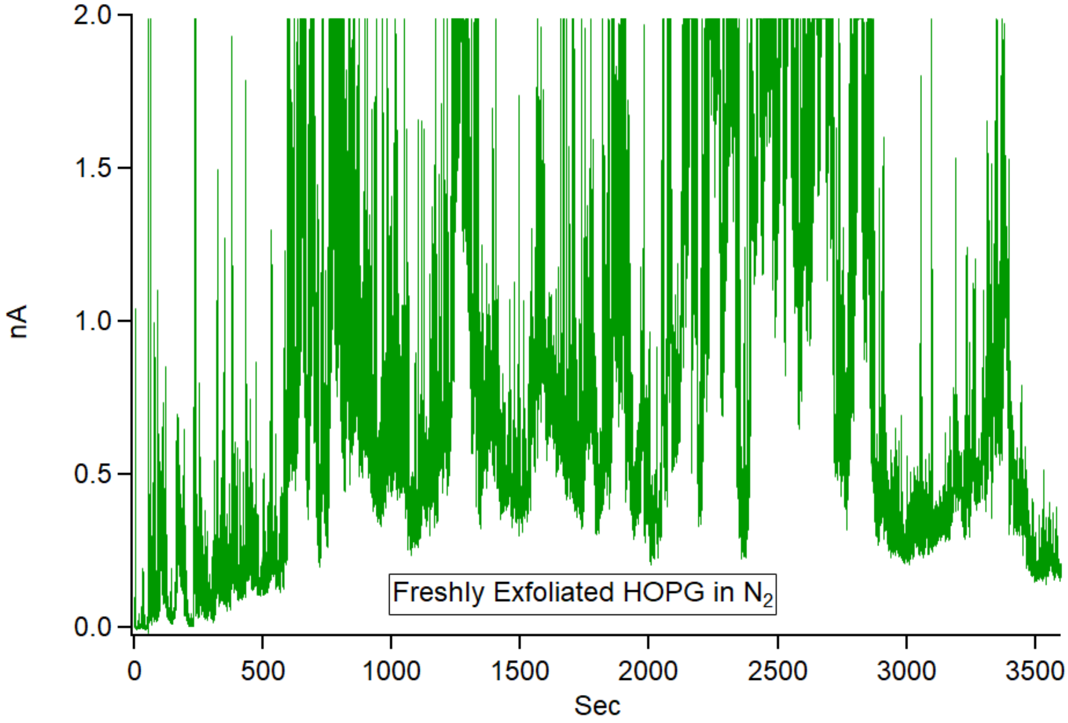

Figure S7. HOPG exfoliated in air and quickly placed in the AFM box which was pumped full of dry nitrogen for about 20 minutes to RH <5% with the probe off of the sample. All data was collected using Cr/Pt coated silicon probes.

Local Spectroscopy Data

Local spectroscopy, I-V curve, data collected in four different conditions, dirty HOPG in air and N_2 and freshly exfoliated HOPG in air and N_2, is shown below. This data was used to calculate average conductances from the slopes of the curves at 0 V over a 100-mV range (except for freshly exfoliated HOPG in air where this range is not possible).

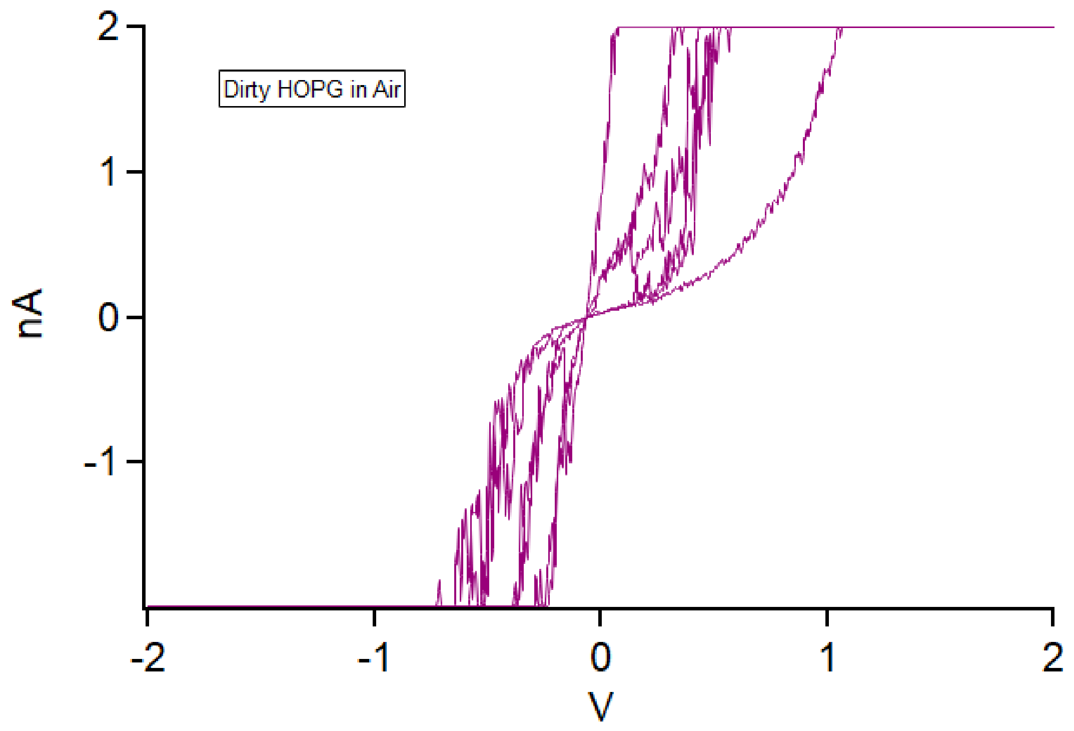

Figure S8. I-V curves taken in open air at ~28 RH on HOPG aged for 5 weeks in a glass vial tipped on its side. All data was collected using Cr/Pt coated silicon probes.

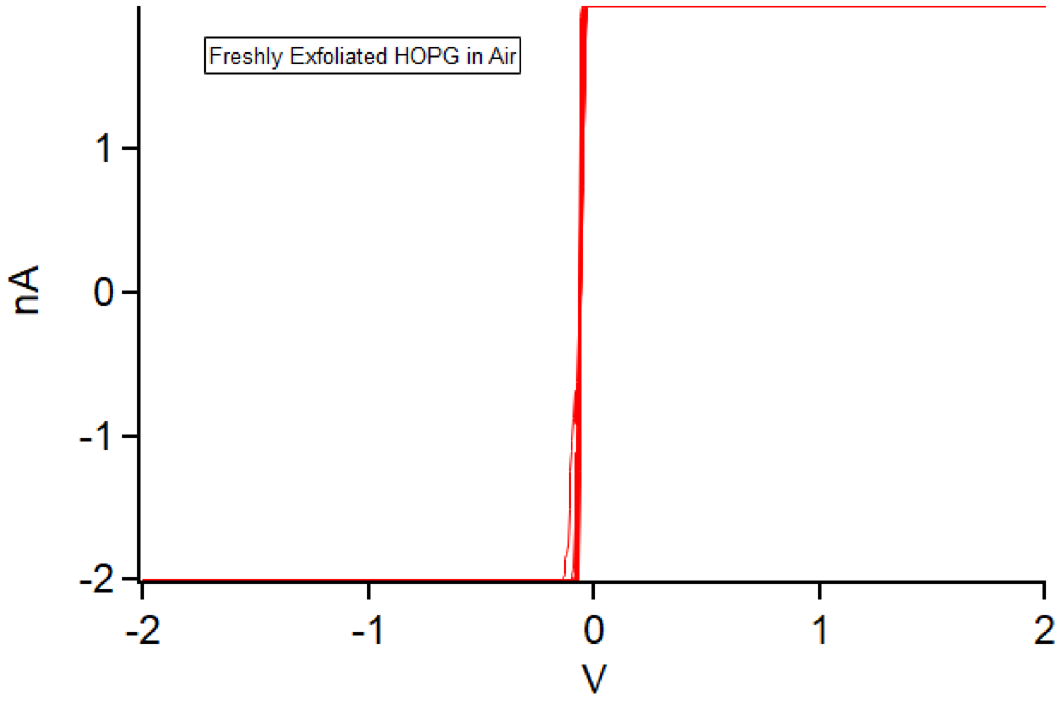

Figure S9. I-V curves taken in open air at ~28 RH on freshly exfoliated HOPG. All data was collected using Cr/Pt coated silicon probes.

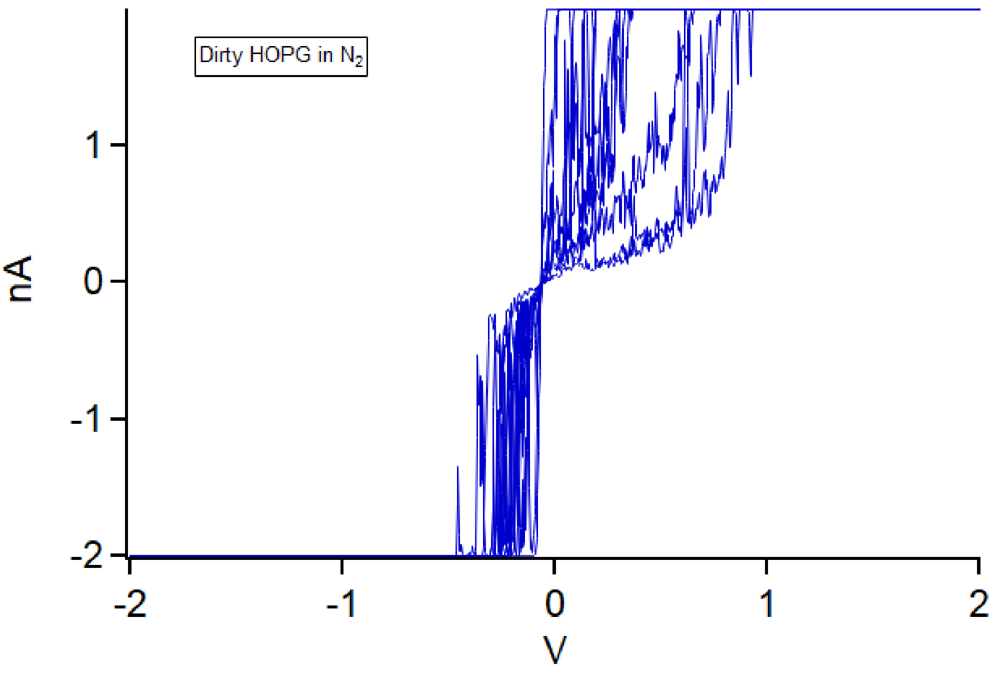

Figure S10. I-V curves taken in open N_2 at ~0.5% RH on HOPG aged for 5 weeks in a glass vial tipped on its side. All data was collected using Cr/Pt coated silicon probes.

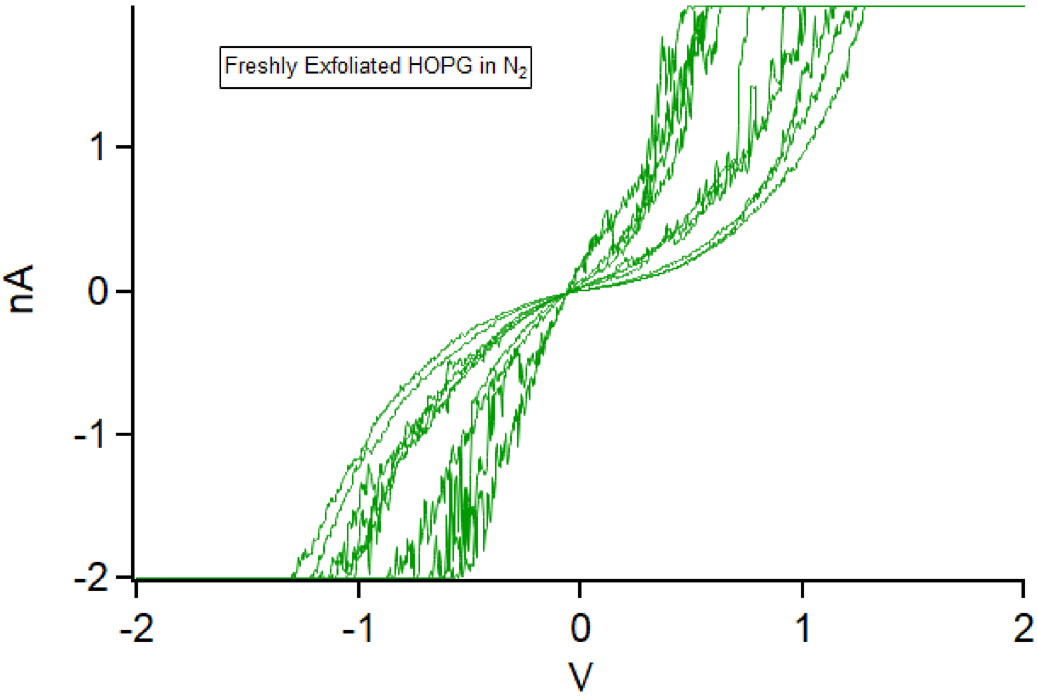

Figure S11. I-V curves taken in N_2 at ~0.5% RH on freshly exfoliated HOPG. All data was collected using Cr/Pt coated silicon probes.

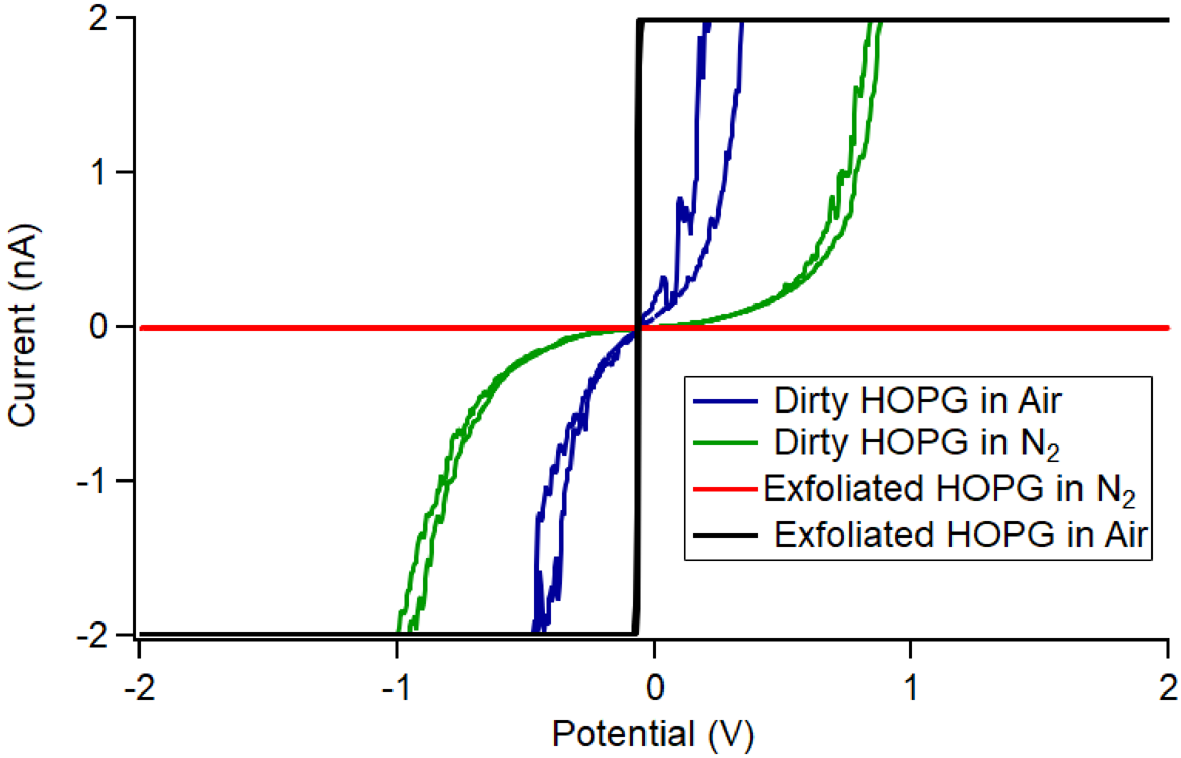

Figure S12. I-V curves taken on exfoliated and 5-week aged HOPG in air (28% RH) and N_2 (1.5% RH) using a CAFM probe made entirely of platinum.

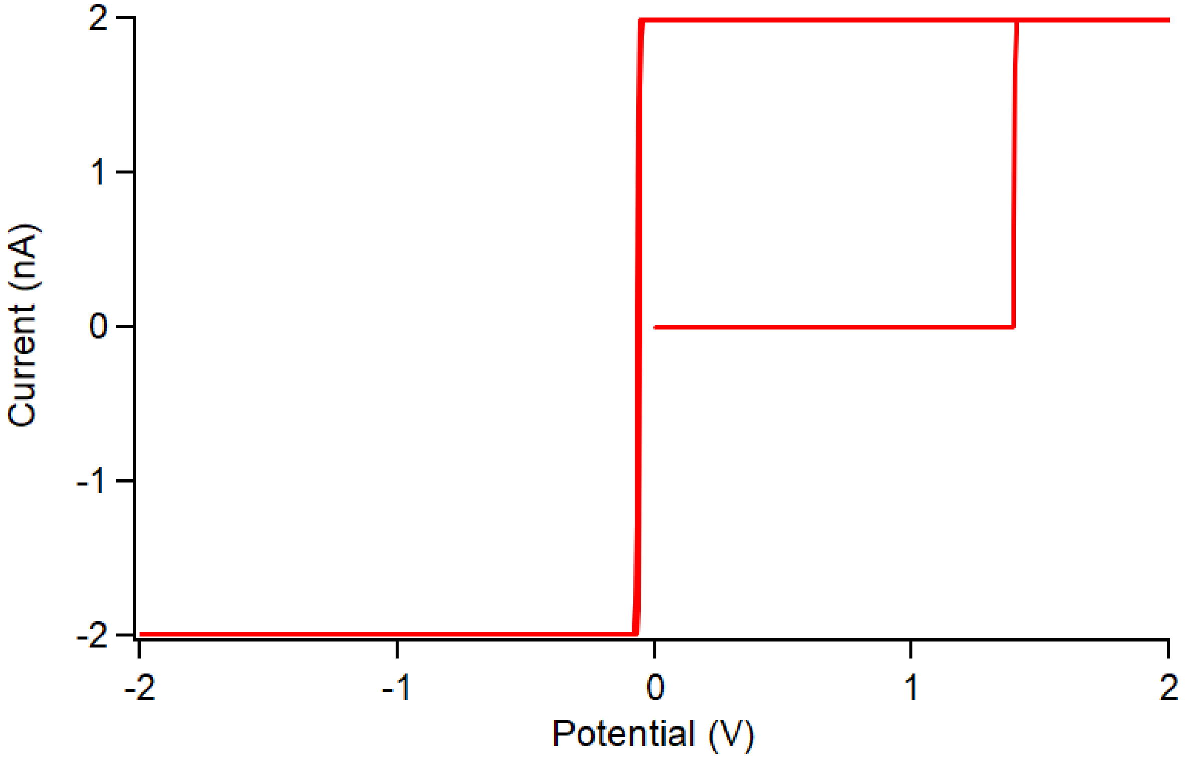

Figure S13. I-V curve taken at very low relative humidity at the point in time when current jumps from noise level to high conductivity. An all-platinum probe was used in this case as well.
